# Cross-Level Mediating Effects of Social Relationships between Religiosity and Successful Aging

**DOI:** 10.1093/geroni/igab046.3337

**Published:** 2021-12-17

**Authors:** Hayoung Park, Hey Jung Jun, Susanna Joo

**Affiliations:** 1 Yonsei University, Seoul, Seoul-t'ukpyolsi, Republic of Korea; 2 Yonsei University, Seodaemun-gu, Seoul-t'ukpyolsi, Republic of Korea

## Abstract

This study aimed to analyze the cross-level mediating effects of social relationships on the association between religiosity and successful aging. The data was the 7th Korean Longitudinal Study of Ageing and the sample was 1,191 couples aged 65 and above. Independent variables were the level of participation in religious activities at the individual level and religious similarity between couples at the couple level. The dependent variable was successful aging at the individual level, which consisted of physical, cognitive, social, and psychological dimensions. Mediating variables were two social relationships: frequency of social interaction at the individual level and marital satisfaction at the couple level. We applied Full Information Maximum Likelihood estimation to include 8% of the sample with missing values in data. According to the multi-level mediation path analysis, both frequency of social interaction and marital satisfaction had mediating effects on the association between the level of participation in religious activities and successful aging; the more they participate in religious activities, the higher the frequency of social interaction and marital satisfaction, and this had positive effects on successful aging. Also, marital satisfaction had a mediating effect on the association between religious similarity and successful aging; when married couples have the same religion, marital satisfaction was higher than when they do not, and this had a positive effect on successful aging. This study is meaningful in presenting multi-dimensional discussions on religiosity and social relationships in later life and a new empirical model to promote successful aging at both individual and couple levels.

